# Validation of a cancer population derived AKI machine learning algorithm in a general critical care scenario

**DOI:** 10.1007/s12094-025-03906-0

**Published:** 2025-03-26

**Authors:** Lauren Abigail Scanlon, Catherine O’Hara, Matthew Barker-Hewitt, Jorge Barriuso

**Affiliations:** 1 Clinical Outcomes and Data Unit, The Christie NHS Foundation Trust, Manchester, M20 4BX UK; 2 Division of Cancer Sciences, Manchester Cancer Research Centre, The University of Manchester, Manchester, M13 9PL UK

**Keywords:** Acute kidney injury, Artificial intelligence, Clinical decision-making, Machine learning, Prospective studies, Retrospective studies

## Abstract

**Purpose:**

Acute Kidney Injury (AKI) is the sudden onset of kidney damage. This damage usually comes without warning and can lead to increased mortality and inpatient costs and is of particular significance to patients undergoing cancer treatment. In previous work, we developed a machine learning algorithm to predict AKI up to 30 days prior to the event, trained on cancer patient data. Here, we validate this model on non-cancer data.

**Methods/patients:**

Medical Information Mart for Intensive Care (MIMIC) is a large, freely available database containing de-identified data from patients who were admitted to the critical care units of the Beth Israel Deaconess Medical Center. Data from 28,498 MIMIC patients were used to validate our algorithm, non-availability of Total Protein measure being the largest removal criterion.

**Results and conclusions:**

Applying our algorithm to MIMIC data generated an AUROC of 0.821 (95% CI 0.820–0.821) per blood test. Our cancer derived algorithm compares positively with other AKI models derived and/or tested on MIMIC, with our model predicting AKI at the longest time frame of up to 30 days. This suggests that our model can achieve a good performance on patient cohorts very different to those from which it was derived, demonstrating the transferability and applicability for implementation in a clinical setting.

## Introduction

Acute Kidney Injury (AKI) is the sudden onset of damage to the kidneys, often caused by another condition, and is when the kidneys suddenly stop working properly. This damage usually comes without warning and can lead to further complications for the patient, increased mortality, and inpatient costs [1]. This is particularly a problem in Oncology patients, who are at high risk of AKI. AKI in these patients can disrupt their treatment, cause further damage to their organs than already caused by their illness, and increase mortality risk [2].

It is important to detect AKI as soon as it occurs and treat the condition before further damage is done. The NHS detection algorithm for AKI is noted to be the most accurate method of detection; current blood creatinine levels are compared to historic values for that patient or a reference range and monitored for an increase to detect AKI [3]; however, this detection occurs when levels of creatinine have risen for the patient, with damage to organs already having occurred. To identify patients at risk of AKI and potentially lessen the impact AKI cause to a patient and disruption to their treatment, our team at the Christie developed a machine learning risk level for upcoming AKI risk for a patient from de-identified blood test results. We used other factors as well as creatinine from routine blood tests conducted at the Christie, to assign a risk level of an AKI within the next 30 days to each patient. Risk levels were categorised from ‘Very low’ to ‘Very high.’ Across a 3 month validation period of the model, an alert of ‘Medium’ risk or higher was triggered in advance of over 60% of actual AKI events and for over 70% of patients that went on to have an AKI [4]. The intent was to create a model that could be utilised alongside the NHS algorithm for AKI detection that would provide an ‘early warning’ that a patient was potentially at risk of AKI, in other that they could be reviewed by the AKI team and mitigations implemented to potentially lessen the risk of avoidable AKI.

This model was developed, trained, tested, and validated at the Christie, a cancer specialist treatment centre, using blood tests routinely done as standard care and gave a good performance. To test robustness, we wanted to test the model on an independent cohort of patients. We looked for a dataset that would enable us to test our model on a vastly different setting and population than the model was trained on, with a greater proportion of AKI and explore any potential barriers to implementation in other care settings.

Medical Information Mart for Intensive Care (MIMIC) [5] is a large, freely available database containing de-identified data from patients who were admitted to the critical care units of the Beth Israel Deaconess Medical Center. There are various versions of these data, MIMIC-IV being the most recent, containing data from patients between 2008 and 2019, collected from Metavision bedside monitors. The MIMIC datasets contain many rich sources of data from the patients’ electronic health records including lab results, vital signs monitoring, and other medical information. These data are freely accessible by researchers and are frequently used to train various machine learning procedures for many purposes, including predicting ICU re-admission, cardiac episodes, septic shock, as well as AKI events [6]. As MIMIC provides such a rich source of data models can be trained and tested and many features evaluated but different approaches can also be evaluated. Xu et al. [7] trained various models on MIMIC-III and stage 1 AKI detected by KDIGO guidelines, evaluating various under and over sampling techniques to address class imbalance.

An alternative usage of the MIMIC dataset is as an external validation of models trained elsewhere, allowing model developers to evaluate their model’s performance on a different cohort of patients to their training set. This allows developers to gather additional evidence for the performance of their model, explore cases in which performance differs and evaluate the availability of the features utilised for their prediction. Zappalà et al. [8] take such an approach, aiming to validate a previously derived machine learning model for the prediction of persistent severe AKI. Utilising data from MIMIC amongst other datasets, they saw good performance to demonstrate scalability of their model, despite lower prevalence of AKI in these validation datasets than their derivation sets and a relatively low sample size.

Our model developed at the Christie was trained on all patients within the hospital, including both inpatients and outpatients, in a population with a much lower prevalence of AKI. We discuss here the process of testing our model on the data contained in MIMIC-IV and validating against AKI detected by an implementation of the NHS detection algorithm and those already identified by KDIGO within the data on MIMIC.

## Materials and methods

Our machine learning model for AKI risk prediction is a Random Forest model, implemented in scikit-learn in Python with derivation described in Scanlon et al. [4]. This was developed utilising routinely collected blood test results from 48,865 patients undergoing cancer treatment at The Christie NHS Foundation Trust between January 2017 and May 2020, to identify AKI events upcoming in the next 30 days. This resulted in an AUROC of 0.881 (95% CI 0.878–0.883) when assessing performance on a test set of 9,913 patients from June to August 2020. The features selected were blood test results from tests routinely done at our institution for all patients, aiming to create a prediction model optimised for our institution. To demonstrate transferability of this approach, this paper describes the evaluation process on data from MIMIC-IV.

The blood test results data used to test our procedure on were accessed from the table “labevents” containing laboratory measurements for patients around the hospital from the database “mimic_hosp", containing information from the hospital wide electronic health record from MIMIC-IV, selecting those blood test results that match the features of our model.

The blood test results contained in the table “labevents" have been collected in different units than those used at the Christie, and so, when accessing these data, we converted the results into the required units for the model. From these comparisons to the mean, median and minimum values for various timescales for urea and creatinine were also derived, which serve as important markers detailing the trends of creatinine and urea values for the patient.

A comparison of the mean and standard deviation between the derivation cohort at the Christie and the MIMIC test cohort is given in Table 1.

**Table 1:** Features included in model and their mean and standard deviation compared to the Christie data

Feature	Unit	Christie		MIMIC-IV	
		Mean	Std Dev	Mean	Std Dev
Current CREA/median over the past year	mol/L	1.01	0.23	1.06	0.46
Total protein (TP) from blood test	g/L	63.9	8.87	66.07	9.68
Current CREA/minimum over the past 7 days	mol/L	1.05	0.17	1.14	0.4
Current CREA—mean over the past year	mol/L	0.03	19.13	−0.16	88.06
Platelet count (PLT) from blood test	× 10^9/L	251.82	143.17	207.62	130.67
Haematocrit (HCT) from blood test	L/L	0.35	0.06	0.32	0.07
Calcium (CA) from blood test	mmol/L	2.27	0.18	2.21	0.2
Lymphocytes (LYMPH) from blood test	× 10^9/L	1.53	6.35	1.66	6.75
Current urea—mean over past year	mmol/L	0.13	1.93	0.12	2.21
Lactate dehydrogenase (LDH) from blood test	IU/L	406.74	541.05	277.53	373
Red cell count (RBC) from blood test	× 10^12/L	3.85	0.73	3.47	0.81
Minimum CREA over the past 7 days	mol/L	72.29	32.01	120.51	137.42
White cell count (WBC) from blood test	× 10^9/L	7.33	9.77	8.29	9.43
Median CREA over the past year	mol/L	74.06	30.39	131.54	135.09
Minimum CREA over the past 2 days	mol/L	74.18	35.02	128.12	143.95
Current CREA—CREA 10 days ago	mol/L	0.02	2.24	0.15	11.42
Creatinine (CREA) from blood test	mol/L	75.11	36.18	136.62	155.1
Current CREA—CREA 20 days ago	mol/L	0.02	1.03	0.14	6.13
Abnormal albumin (ALB) 1 if 35 ≤ ALB ≤ 50	1, 2 or 3	1: 80.1%		1: 60.7%	
2 if ALB ≤ 34		2: 19.6%		2: 38.9%	
3 if ALB ≥ 51		3: 0.3%		3: 0.4%	
Current CREA—minimum over the past 2 days	mol/L	0.93	5.35	8.5	29.76

Most features involve values as they appear in the blood test result for the patient, but some were calculated as comparisons to mean, median, and minimum values over a given time period for that patient. These include calculations of the following ratios; ratio of the current creatinine value over the median from the past year, ratio of the current creatinine value over the minimum from the 7 days and the following differences; the difference between the current creatinine value and the mean over the past year, the difference between the current urea value and the mean over the past year, the difference between the current creatinine value and the value 10 days ago, the difference between the current creatinine value and the value 20 days ago, and the difference between the current creatinine value and minimum value over the past two days

AKI events are routinely detected at the Christie using the NHS detection algorithm implemented in SQL for immediate detection of AKI from blood test results. We used this same procedure to detect AKI using the creatinine results in database “mimic_hosp" and table “labevents" and patient gender and reference age from the database “mimic_core" table “patients". We calculated age at time of blood test from the year of the blood test, and the “anchor_age" and “anchor_year" given.

**Fig. 1:**
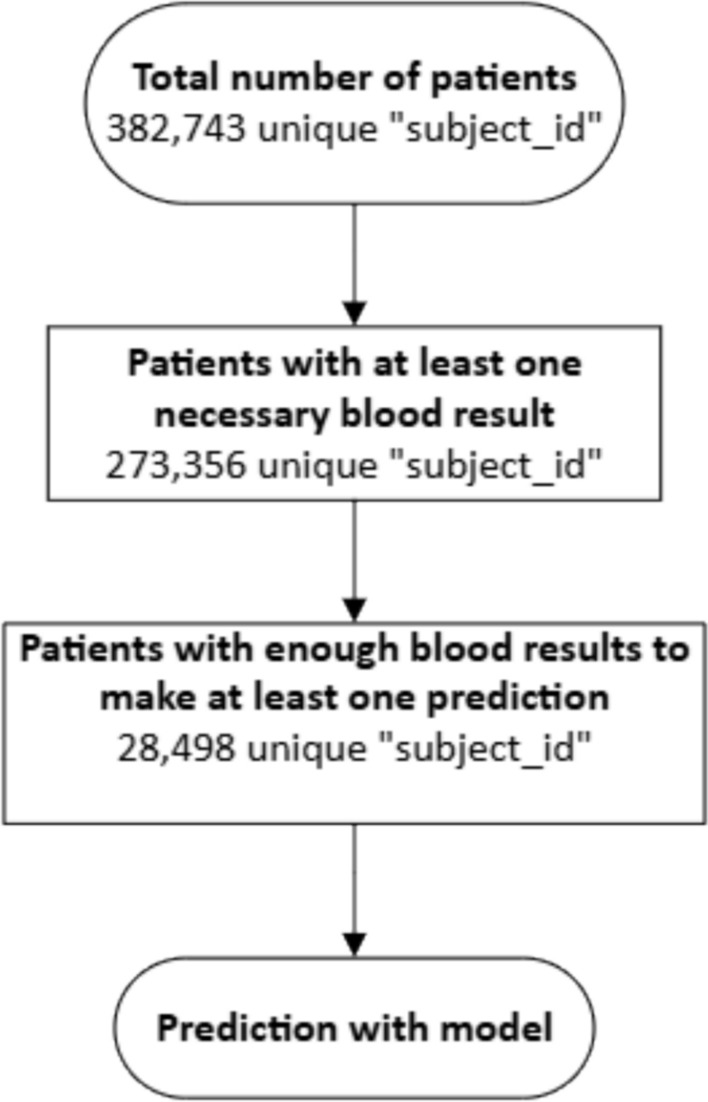
Flow diagram detailing the patients incorporated in the model testing. The only exclusion criteria were availability of blood results needed to make a prediction which resulted in 28,498 patients. The largest removal criteria were the availability of a measurement of Total Protein for the patient

The inclusion of patients is given in Fig. 1. All patients with blood test results available in the MIMIC-IV “mimic_hosp" database were included in this study providing that they had the necessary blood results to make a prediction. The vast majority of blood results necessary for the model to generate a prediction were available for the majority of patients; however, many patients did not have a measure of Total Protein which is necessary for the model to make a prediction. 273,356 patients had at least one necessary blood result to be considered for inclusion in this study; however, 225,158 did not have a Total Protein blood result at any point and so were not included in the study. This left 28,498 patients to be included in this study with the available blood results to make at least one AKI prediction.

The data were extracted from the MIMIC-IV database using Google BigQuery, with the prediction model tested in scikit-learn in Python. The main metric of comparison to other published work is the AUROC as this is the most widely reported metric by authors; however, in comparison to the validation of the model internally at the Christie, we also report the percentage of AKI identified before occurrence and the percentage of patients with AKI identified.

## Results

**Fig. 2:**
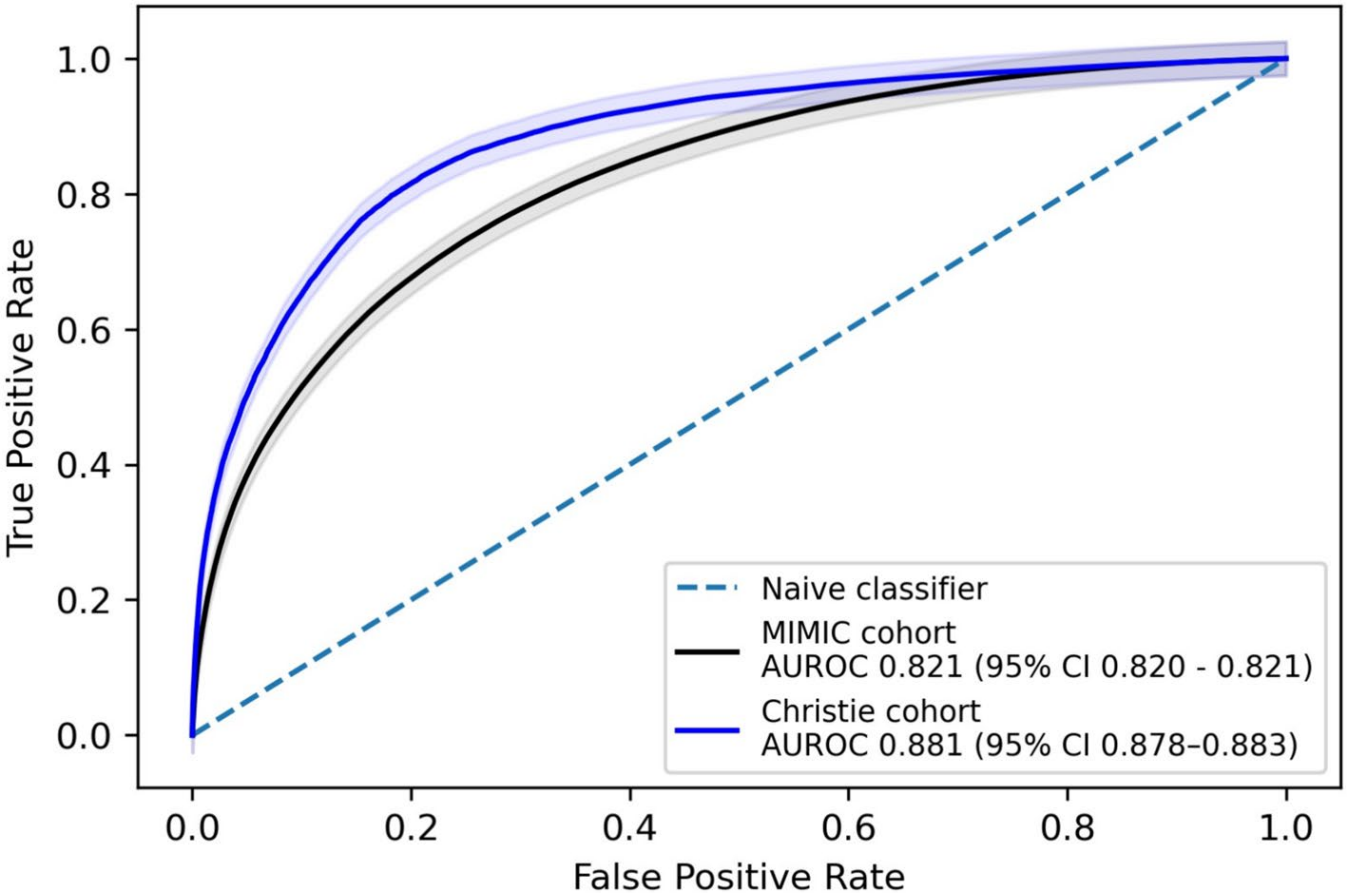
A comparison of AUROC curves for the Christie test population and the external MIMIC test cohort. Whilst performance on the external MIMIC test cohort was slightly poorer than the performance on the Christie test set, it still represents good performance on this external test cohort

Assessing predictions per blood test resulted in an AUROC of 0.821 (95% CI 0.820–0.821) which, whilst slightly poorer than the performance on the Christie test set of 0.881 (95% 0.878–0.883), represents good performance on this external test cohort, with both AUROCs shown in Fig. 2.

Assigning risk levels using the same classifications as described in Scanlon et al. [4] resulted in 67.3% AKI occurrences being allocated a ‘Medium’ or higher risk level at least once within 30 days before occurrence, with 86.6% of patients receiving a ‘Medium’ or higher risk level at least once before at least one of their AKI occurrences.

Per blood test, these risk levels result in a sensitivity of 0.431 and specificity of 0.935 for a ‘Medium’ or higher risk level for identification of an AKI within 30 days of that blood test for the patient.

We also evaluated the model against the KDIGO stages given in the table “kdigo_stages" in the database “mimic_derived" resulting in an AUROC of 0.752.

In comparison to the performance of other models defined and tested on MIMIC, reported in Tables 2 and 3, we see the performance of our model tested on MIMIC is in line with the performance others report with our model having the largest window for prediction. Whilst we note that only 28,498 patients could be included in the prediction process, this is not the lowest number of patients when compared with other predictive work and, in fact, is in about the middle of all sample sizes.

**Table 2:** Comparison of our model to others

Authors	AKI detection criteria	Best model
Pan et al. [9]	RIFLE	Self-correcting RNN with regularisation
Wang et al. [10]	From label in dataset	CNN
Sidney et al. [11]	KDIGO stage 2 or 3	CNN
Zimmerman et al. [12]	KDIGO	Logistic regression
Mohamadlou et al. [13]	NHS stage 2 or 3	XGBoost
Mohamadlou et al. [13]	KDIGO stage 2 or 3	XGBoost
Wang et al. [14]	Not explicitly stated	XGBoost
Li et al. [15]	KDIGO	L2 regularized logistic regression
Yue et al. [16]	KDIGO	XGBoost
Zhang et al. [17]	KDIGO	XGBoost
Luo et al. [18]	Not explicitly stated	XGBoost
Scanlon et al. [4]	NHS	Random forest
Scanlon et al. [4]	KDIGO	Random forest

NB AUROCs are over different timescales and sometimes assessed on hold out test sets or through cross validation, we are reporting the best AUROC reported in the paper. Some papers have multiple models discussed; in this table, we include only the reported best-performing model

**Table 3:** Comparison of our model to others

Authors	Number of patients included	Best AUROC	Time before onset
Pan et al. [9]	Around 25,000	0.893	6 h
Wang et al. [10]	2,663	0.984	Not explicitly stated
Sidney et al. [11]	12,347	0.856	48 h
Zimmerman et al. [12]	23,950	0.783	72 h
Mohamadlou et al. [13]	48,582	0.749	12 h
Mohamadlou et al. [13]	48,582	0.914	12 h
Wang et al. [14]	46,593	0.95	24 h
Li et al. [15]	14,470	0.779	72 h
Yue et al. [16]	3,176	0.817	Not explicitly stated
Zhang et al. [17]	18,186	0.880	24 h
Luo et al. [18]	15,218	0.849	72 h
Scanlon et al. [4]	28,498	0.821	Up to 30 days
Scanlon et al. [4]	28,498	0.752	Up to 30 days

NB AUROCs are over different timescales and sometimes assessed on hold out test sets or through cross validation, and we are reporting the best AUROC reported in the paper. Some papers have multiple models discussed; in this table, we include only the reported best-performing model

**Fig. 3:**
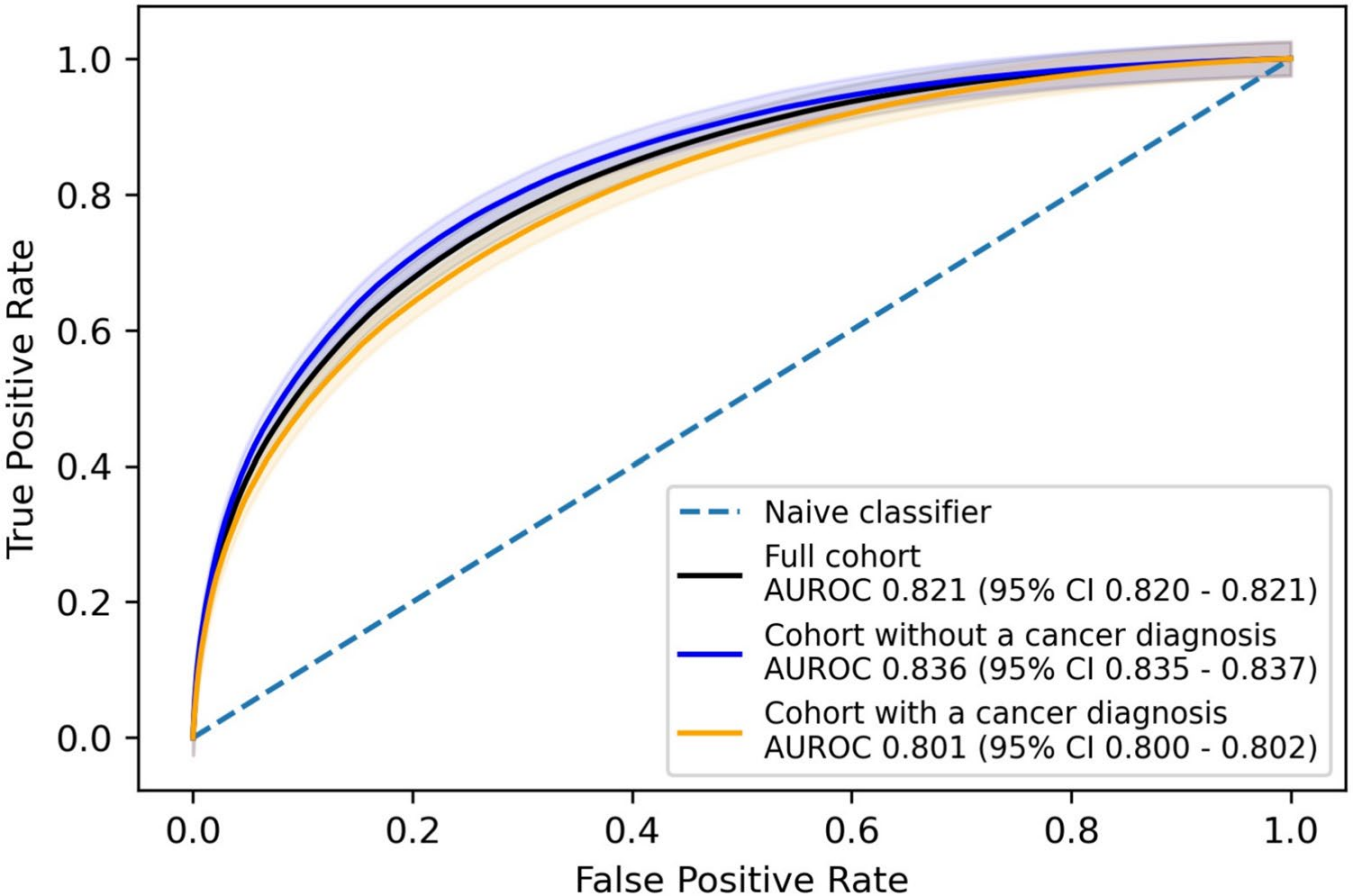
A comparison of AUROC curves for those with a cancer diagnosis and no cancer diagnosis from the MIMIC cohort, compared to the full cohort

Using the database “mimic_hosp" and table “diagnoses_icd" from MIMIC-IV, we gather ICD9 and ICD10 codes to identify which of the 28,498 patients had a cancer diagnosis. We identify those ICD9 codes between 140 and 239 and ICD10 codes between C00 and D49 as related to cancer [19, 20]. This table includes an identifier for the patient, ‘subject_id,’ and an identifier for the hospital stay it relates to, ‘hadm_id.’ 11,704 of the 28,498 had an ICD code related to cancer for at least one of their hospital stays. As patients may have had multiple blood test results and multiple hospital stays, we use the ‘hadm_id’ to determine if a patient had a cancer diagnosis on or before the blood test. For blood tests that were for patients after a cancer diagnosis, we get an AUROC of 0.801 (95% CI 0.800–0.802) and for those that were before a cancer diagnosis or no diagnosis was recorded for the patient an AUROC of 0.836 (95% CI 0.835–0.837), with both AUROCs and a comparison to the full cohort shown in Fig. 3.

## Discussion

The aim of this study was to test our AKI prediction model outside of its “comfort zone", a solely cancer population. Our prediction model was built on data from both inpatient and outpatient cancer patients of all ages treated at the Christie NHS Foundation Trust. Our model was designed to predict occurrence of AKI within the next 30 days with a high level of success and the next step was to test the model on an external cohort of patients. The MIMIC_IV data was chosen as it includes only patients admitted to ICU and has a much higher prevalence of AKI as a whole than on the derivation cohort at the Christie, which presents a “stress test" to The Christie algorithm as it was not developed in an acute clinical setting.

Using data from all patients in the MIMIC-IV data that had the relevant blood results with no other exclusion criteria. Our model realised an AUROC of 0.821 and identified 67.3% of AKI occurrences for 86.6% of patients with AKI. This performance, on a completely different cohort to the one it was derived from, is similar to that achieved on the original Christie dataset, thereby demonstrating the robustness of the model. We investigated cancer diagnoses in the 28,498 patients included in this study and saw only a slight difference in AUROC when splitting these patients into two groups, those blood tests after a cancer diagnosis, and those with no prior cancer diagnosis, 0.801 and 0.836, respectively, further demonstrating model robustness.

This translation of a model and maintained performance is similar to that seen by Mohamadlou et al. [13] and Wang et al. [14] when they tested models derived elsewhere on data from MIMIC. However, in these cases, the training cohorts were similar to the MIMIC test cohort, with Wang et al.’s model trained on a data source of ICU stays in China, and whilst Mohamadlou et al.’s derivation set was not solely ICU stays but all inpatient hospital stays, ours still differs in that it included both inpatients and outpatients of all ages at a specialist cancer treatment centre. We find it very interesting that a model derived in such a different cohort of patients can achieve such good performance on an external test cohort. This could be linked to the fact that the original model was developed in an agnostic manner including a wide variety of patients from follow-up cancer patients to palliative ones. The two clinical scenarios ICU and cancer population in any situation (active treatment up to follow-up) differ in multiple aspects. The fact that the algorithm works on both settings could be an indication that agnostic development of predictors have the power of overcome known clinical differences using “hidden" patterns from “unused" data. Zappalà et al. [8] also result sustained performance when testing their model defined elsewhere on data from MIMIC but utilised a much smaller sample size for this test cohort than our test, due to the exclusion criteria necessary to test their model. Although our exclusion criteria reduced the total patient sample size to 28,498 patients who had the necessary blood tests for prediction, its sustained performance on this relatively high sample size compared to others gives confidence to its transferability.

It is encouraging that when evaluated against other models derived and tested on MIMIC, our model achieves an AUROC of 0.821, which is in line with the other models derived and tested on this source (Tables 3 and 4) especially when it is considered that our model predicts AKI at the longest time frame of up to 30 days. This time frame was derived to fit with patient treatment schedules at the Christie and allow for AKI to be predicted in enough time to intervene especially when considered it may be picked up in outpatient appointments. The window may not need to be as large for those patients in ICU; however, it is encouraging that, despite this, performance remains high.

When evaluated against AKI detected by KDIGO, the AUROC was lower than the AUROC for AKI detected by the NHS detection algorithm. We note that for AKI defined by KDIGO, the prediction model over predicts AKI occurrences, which is unsurprising as the NHS algorithm is noted to detect AKI sooner than KDIGO guidelines; however, there is some criticism that this detection can be over sensitive.

This study further demonstrates how our model may perform in other organisations. Although many of the patients included in MIMIC-IV could not be incorporated into our model test due to missing blood test results, when compared to the cohort size of other models derived and tested on the same data, our sample size of 28,498 patients is amongst the average sample size for studies of this type and is higher than the sample size used for various other models validated on MIMIC (Tables 3 and 4).

The only limitation for our model being utilised more widely is the availability of a measurement for ‘Total Protein’ which was the largest removal criteria for patients within MIMIC. Our model was derived to be utilised within the Christie where these features are available for every patient receiving routine blood tests. If we had trained the model on MIMIC, optimising for features available in those data, different features may have been selected but in turn may have resulted in a model not necessarily transferable to utilising the model in practise. Despite this, performance is maintained on this reduce sample size when compared to our original derivation cohort and compared to alternative AKI prediction models, suggesting that those interactions between other blood results identified by our agnostic model development are consistent in different populations.

Overall, we are encouraged to conclude that despite the occurrence of AKI being much lower in our derivation cohort from the Christie than the MIMIC test cohort, the model still achieved good performance on the MIMIC data. This paves the way for future work in which we would like to extend the testing of our model and start to implement intervention strategies for AKI upon prediction.

## Conclusion

The translation of a model derived in a cancer population on both inpatients and outpatients to a varied cohort of critical care inpatients for the prediction of AKI demonstrates a stress test of this model, with performance comparable to models derived and tested solely on the MIMIC data sources. The benefits of deriving the model elsewhere then testing on MIMIC allow for the assessment of the availability of features utilised in the model and the exploration of performance on cohorts where the prevalence of AKI differ to the derivation cohort.

The model achieved good performance when tested on this external data source, demonstrating model robustness and giving motivation to the implementation of this model.

## Data Availability

The de-identified dataset MIMIC-IV analysed for this study is available at the following link: https://physionet.org/content/mimiciv/3.1/
